# Practical Prescriptions

**Published:** 1904-10

**Authors:** 


### PRACTICAL PRESCRIPTIONS.

---

AN EFFICIENT GALACTAGOG.

℞ Glycerole of the compound acid glycero-phosphates (Huxley) .................................... ℥iv.
Distilled water, add .................................... ℥xvi.

M. F. Solut. Sig.—One tablespoonful four times daily.

Each dose contains exactly four grains of the acid glycerophosphates of soda, lime, potash, iron and manganese, in the exact physiological proportions laid down by Huxley, with a 250th part of a grain of strychnine.

It is pleasant to take and is the most reliable of the acid glycero-phosphate preparations.

Under the influence of this solution and suitable diet, the lacteal secretions are increased without fatigue to the mother. The treatment should be continued during lactation for the first three months at least.

---

ANALGESIC SALVE FOR RHEUMATISM

TO RELIEVE LOCAL PAIN.

℞ Lanoline (anhydrous) .................................... ℥i.
Menthol .................................... ʒi.
Betul-ol (methyl-oleo-salicylate comp.) .................... ℥i.
Beeswax .................................... ʒi.

Misce fiat unguent. secundum artem.

Apply with friction where it is not too tender, or on lint covered with oil silk if very sensitive to rubbing. It gives almost immediate relief.

FOR ACNE OF THE FACE.

| | | |
|---|---|---|
| ℞ | Betul-ol (methyl-oleo-salicylate comp. | 1 gram. |
| | Ung. storax | 1 " |
| | Lanoline (anhydrous) | 20 " |

M. F. Ung.

Apply every night with friction for ten days; then interrupt the treatment. Recommence after ten days again if necessary.

GOUTY ECZEMA.

LUFF recommends liq. plumbi for this very obstinate trouble. The following modification will be found very soothing, especially if followed by a simple dusting powder of starch and talc powder.

| | | |
|---|---|---|
| ℞ | Liq. plumbi subacetatis | ʒi. |
| | Hux-sal (antiseptic salt) | ʒi. |
| | Aquæ sambuci ........ad | ℥xvi. |

M. Ft. Applic. Sig.—For external application over the parts affected. Internally, one capsule of colchi-sal every two hours.

FOR SCABIES.

| | | |
|---|---|---|
| ℞ | Icthyol | ʒiv. |
| | Hux-sal (antiseptic salt) | ʒs.s. |
| | Glycerini | ʒiv. |
| | Aquæ distil | ʒv. |

M. F. Applic. Sig.—Apply externally two or three times daily.

